# Silver Halide-Based Nanomaterials in Biomedical Applications and Biosensing Diagnostics

**DOI:** 10.1186/s11671-022-03752-x

**Published:** 2022-11-28

**Authors:** Lin Zhang, Hong Zhang

**Affiliations:** Shandong University of Traditional Chinese Medicine Affiliated Hospital, No. 16369, Jingshi Road, Jinan, 250014 Shandong People’s Republic of China

**Keywords:** AgX nanomaterials, Photodynamic therapy, Antibacterial, Biosensors

## Abstract

In recent years, silver halide (AgX, X = Cl, Br, I)-based photocatalytic materials have received increasing research attention owing to their excellent visible-light-driven photocatalytic performance for applications in organic pollutant degradation, HER, OER, and biomedical engineering. Ag as a noble metal has a surface plasma effect and can form Schottky junctions with AgX, which significantly promotes electron transport and increases photocatalytic efficiency. Therefore, Ag/AgX can reduce the recombination rate of electrons and holes more than pure AgX, leading to using AgX as a photocatalytic material in biomedical applications. The use of AgX-based materials in photocatalytic fields can be classified into three categories: AgX (Ag/AgX), AgX composites, and supported AgX materials. In this review, we introduce recent developments made in biomedical applications and biosensing diagnostics of AgX (Ag/AgX) photocatalytic materials. In addition, this review also discusses the photocatalytic mechanism and applications of AgX (Ag/AgX) and supported AgX materials.

## Introduction

Among various types of inorganic nanoparticles (NPs), silver NPs (AgNPs), due to their unique chemical, physical, and biological properties, have received a lot of attention for biomedical and bioanalytical applications [1–5]. For example, AgNPs are well known for their highly effective antimicrobial activities [2, 4, 6, 7] both in solutions and in solids. In addition, AgNPs have been widely used in several areas, such as sterilization [8–10], iconography, [11–13] water treatment [14–16], food package [17–19], agriculture [20–23], medicine [24–27], photocatalysis [28, 29], biotechnology [30–32], cancer therapy [33–37], Raman spectroscopy [38–40], electrochemistry [41–44], and environmental monitoring [44–47].

AgNPs can be combined with other nanomaterials to improve their performance. For example, AgNPs can be reduced on some semiconductors (such as TiO_2_, ZnO, and WO_3_) to form the Schottky junction [33, 48–50]. AgNPs have also been combined with magnetic NPs [51], graphene oxide [52], and quantum dots for various applications [53].

Silver halides have been widely used as industrial catalysts and photosensitive materials [54–61], and the use of silver halides is discussed in the following part. Chemically, silver has a strong affinity with halides to form various AgX (X = Cl, Br, and I) materials. There are numerous methods to prepare Ag/AgX. For example, AgX is doped on the surface of AgNPs by exposure to light of appropriate wavelengths, high-temperature reduction, and reducing agents. Ag/AgX shows better activity and selectivity toward certain applications. Thus, Ag/AgX as a nanocomposite has been extensively researched in recent years [8, 54, 62–68].

Since silver chloride has very low solubility in water, it is often used in the laboratory to determine the silver content of samples. Another very important application of silver chloride in electrochemistry is to make silver/silver chloride reference electrodes [69]. AgBr has been applied in several areas [70], such as color-changing lenses, in which a very small amount of silver bromide and copper oxide were added to ordinary glass. AgBr has photosensitivity and is often used in photographic films. A thin layer of gelatin containing fine AgBr NPs is coated on printing paper. When light with different intensities hit the film, AgBr decomposition products of different degrees are produced on the film. The more AgBr breaks down, the more darkness on that part. At last, AgI can condense water vapor in the air and form rain. Thus, AgI has been used as a nucleating reagent to precipitate supercold clouds and induce artificial rain. Aside from the above traditional applications, the photosensitivity of AgX has been recently utilized in biomedical and bioanalytical applications [71–73].

Although AgX can be applied in numerous ways, there are still some challenges and limitations. For example, AgX responds to ultraviolet and visible light, but light in this wavelength range cannot penetrate most human tissues. When applied to clinical studies, the toxicity of AgX has not been thoroughly studied. In some photocatalytic systems, photocorrosion of AgX can occur. Over the last few decades, a large surge in research activities emerged for biological applications of nanomaterials. Among them, the use of AgX has been growing most rapidly. Most related works were published in the last 15 years. In this article, we review the current status of the field with a focus on anticancer, antibacterial applications, and bioanalytical sensors.

## Photocatalytic Activities of AgX

Before reviewing examples of various applications, we first describe the mechanism of photocatalytic activities, which are the basis for the design of materials and their properties. The principle of photocatalysis is based on the redox activity of photocatalysts under light to achieve degrading pollutants, material synthesis, and chemical transformations. Normally, photocatalytic oxidation uses semiconductors as catalysts and light as energy to degrade organic matter into carbon dioxide and water, where advanced oxidation by TiO_2_ is a primary example [48, 56, 74–76].

AgX belongs to semiconductors, and the bandgaps of AgCl, AgBr, and AgI are 3.28, 2.89, and 2.22 eV, respectively [77]. Pure AgX as a semiconductor has a low photocatalytic efficiency under ambient light irradiation [78–80]. Nevertheless, Ag nano-dots can still swiftly grow on the surface of AgX even with weak light irradiation [8, 54, 65–67, 69], which generates Ag/AgX nanocomposites.

**Fig. 1:**
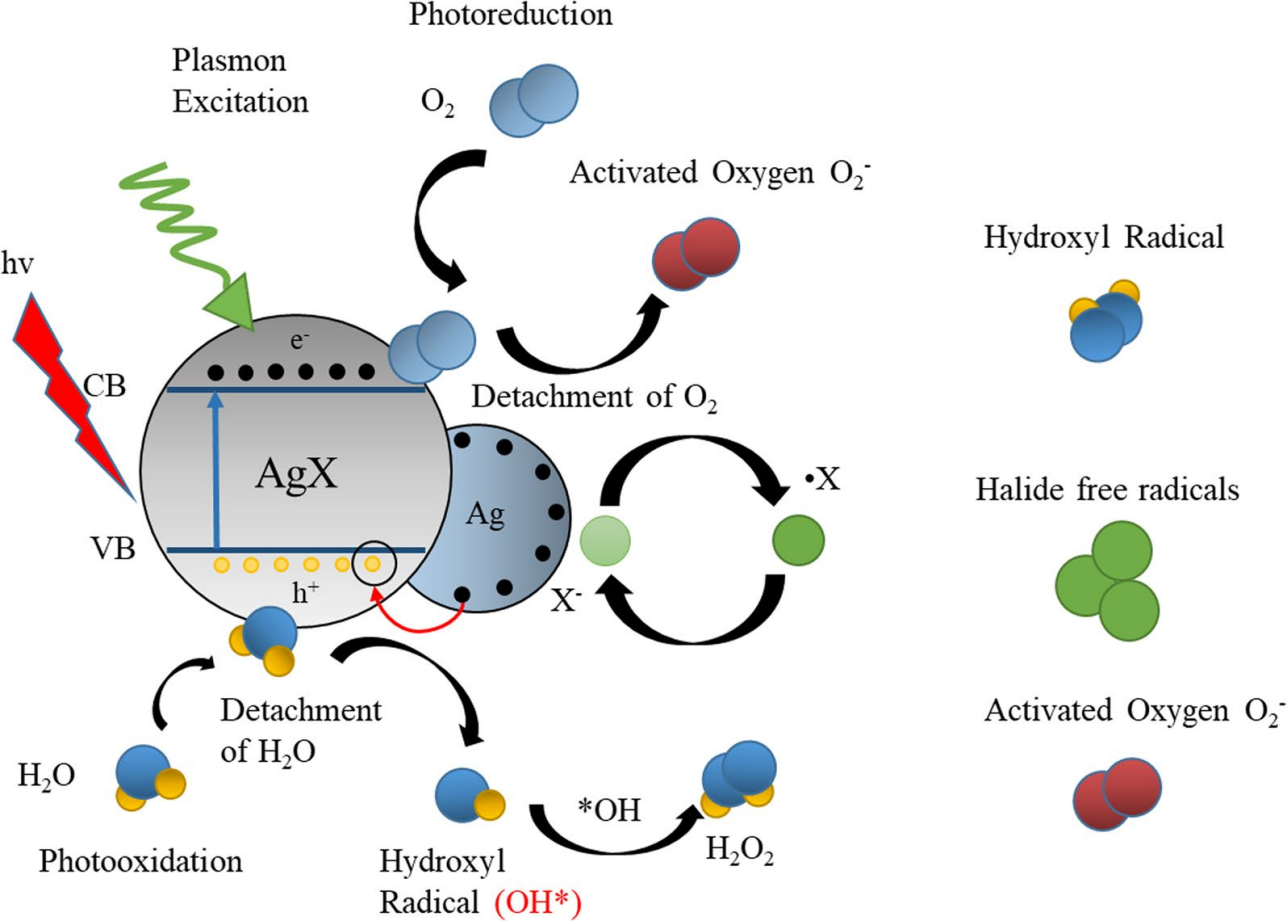
The photocatalytic mechanism of AgX and AgNPs. The AgX as a semiconductor absorbs photons under light irradiation, which generates photoelectron–hole pairs when the energy is greater than the bandgap. The photocarriers migrate to the surface under the action of the electric field or by diffusion motion. The electrons and holes migrate to the surface of the catalyst and react with the adsorbed material on the surface of the catalyst, respectively. Meanwhile, Ag as a conductor can promote electron transfer to the holes on AgX NPs

The photocatalytic mechanism of Ag/AgX nanocomposites is displayed in Fig. 1. The Ag metal as a conductor grown on the surface of AgX can easily generate electron–hole pairs, and then those electrons in the valance band can absorb the photons with energy equal to or higher than the bandgap energy. Those photo-generated charge carriers can only be deactivated by O_2_ or H_2_O.The first process is the photo-generated charge-mediate redox reactions, which include the oxidation of H_2_O to form OH^·^ radicals, and the reduction of O_2_ to form reactive oxygen species (e.g., singlet oxygen, hydroxyl radical, superoxide, hydroperoxyl radical, and hydrogen peroxide) [81, 82]. This is the main process that can generate ROS. The second process is deactivation, which produces electron–hole pairs on the other side and recombines to obstruct redox reactions. Therefore, the second process decreases the efficiency of photocatalysis, which explains why pure AgNPs have a weak photocatalysis activity.

To inhibit the second process, that photo-generated charge recombination can be decreased by adding a semiconductor photocatalyst, such as AgX. AgX coated on AgNPs would naturally form a Schottky junction, which is supposed to trap the photo-generated charge carriers and then provide free electrons to minimize the recombination process [83–87]. Those electron–hole pairs generated by photo-excitation would migrate from AgNPs to the conduction band of AgX. In other words, these excess electrons in the semiconductor (AgX) side will migrate to holes in the metal side (Ag) through the Schottky junction.

Meanwhile, a large number of electrons generated by AgNPs can accelerate the process of photocatalysis, because of the localized surface plasmon resonance (LSPR) of AgNPs [57–59, 88–90]. Noble metal NPs, such as Au, Ag, Pt, and Cu, exhibit strong UV–Vis absorption due to their surface plasmon resonance (SPR). When AgX is decomposed into Ag^0^, the nanocomposite can demonstrate strong SPR and enhance the photocatalytic ability. As a result, plasmonic NPs can serve as an alternative type of sensitizer to enhance the visible light absorption of photocatalysts without the problem of degradation like organic sensitizers [91–93]. In 2008, Wang et al. fabricated Ag/AgCl plasmonic photocatalysts by an ion-exchange method [58], which triggered an upsurge in researching Ag/AgX plasmonic photocatalysts.

## AgX for Cancer Therapy

Cancer has the biological characteristics of abnormal cell differentiation and proliferation, loss of control of growth, invasion, and metastasis. In 2020, an estimated 19.3 million new cancer cases and almost 10.0 million cancer deaths occurred worldwide. The most common cancers are esophagus, lung, stomach, breast, and cervical cancers. According to the analysis, the global cancer burden would increase by 28.4 million cases in 2040, a 47% rise from 2020. Meanwhile, a larger increase in developing (64% to 95%) countries will occur than in developed (32% to 56%) countries [94]. Therefore, nanotechnology in cancer treatment and diagnosis has gained popularity because it may have fewer side effects than traditional chemotherapeutics, and radiation therapy, which can generate cytotoxicity to normal cells as well [95–101]. To enhance the therapeutic effect and decreases the side effects, photodynamic therapy (PDT) is widely used in cancer treatment today [102–110].

AgX-based nanostructures have high biocompatibility, photocatalytic ability, and low toxicity, which have been regarded as attractive candidates to increase the clinical therapeutic effect of numerous kinds of cancer such as skin cancer and mouth cancer [33, 68, 96, 111]. Due to the limitation of effective wavelength, most current organic photosensitizers (less than 600 nm) cannot be used for tissues thicker than 0.1 cm since light cannot reach them. In addition, these organic photosensitizers are sometimes difficult to be excreted from the body. The wavelength range of AgX photosensitizers can be adjusted, and the application of a far infrared band can produce photothermal and photodynamic combined therapy. Since the particles can become smaller, they can be metabolized by the human body. In the following section, we review representative studies of Ag/AgX nanostructures in targeted drug delivery and controlled release systems.

### Photodynamic Therapy

In PDT, the tumor site is irradiated under the light of specific wavelengths to activate photosensitive drugs, selectively concentrate them in the tumor tissue, and trigger photochemical reactions to destroy the tumor. The superoxide disproportionation reaction occurs under light irradiation. Photosensitive drugs can generate ROS (hydroxyl free radicals and superoxide free radicals), which can produce cytotoxicity and kill tumor cells. PDT has the advantage of delivering precise and effective treatments with fewer side effects than traditional cancer therapies.

**Fig. 2:**
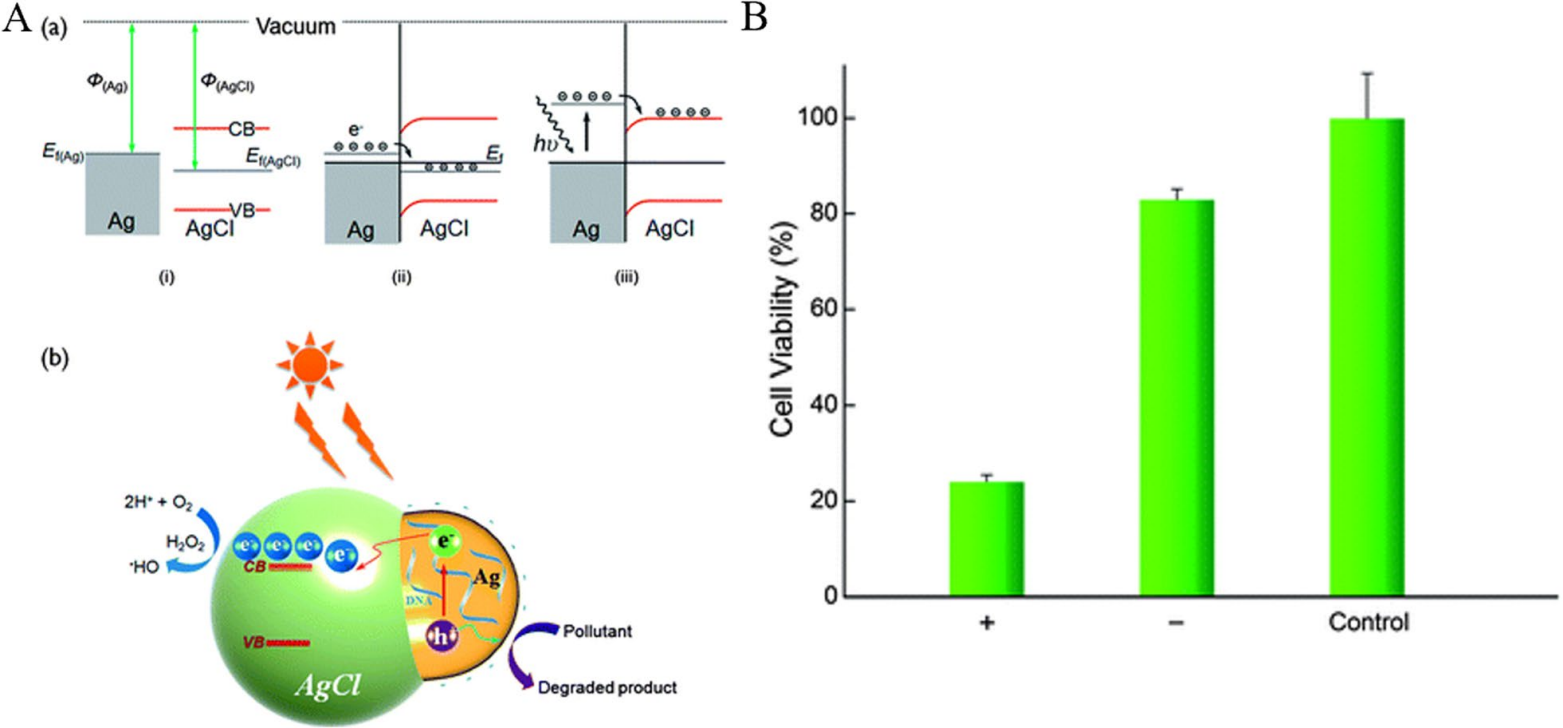
**A** Illustration of the plasmonic Ag/AgCl nanostructure-based photocatalytic reaction mechanism. The DNA capping makes silver nanostructures negatively charged. **B** A comparison of viability among HeLa cells incubated with (+) and without (−) the Ag/AgCl nanostructures in PBS after photo-irradiation for 8 min. Adapted from Ref. [111] with permission. Copyright (2013), Journal of Materials Chemistry B

Wang et al. reported DNA-templated plasmonic Ag/AgCl nanostructures in 2013 [111], which have selective photocatalysis and photocatalytic ability for killing cancer cells. They introduced DNA-programmable synthesis of ∼20 nm Ag/AgCl nanostructures by short-term (5 min) UV irradiation as shown in Fig. 2A, yielding monodispersed AgNPs in the presence of DNA. Moreover, these optimal DNA-encapsulated structures show DNA sequence-specific sizes down to less than 40 nm with an Ag/AgCl composition ratio of 2: 1 that afforded a vastly increased surface area and higher photocatalytic activity. The cell counting kit-8 (CCK-8) cytotoxicity assay showed a decrease of around 75% in the viability of the photocatalyst-treated HeLa cells with 532 nm light irradiation for 8 min (Fig. 2B).

**Fig. 3:**
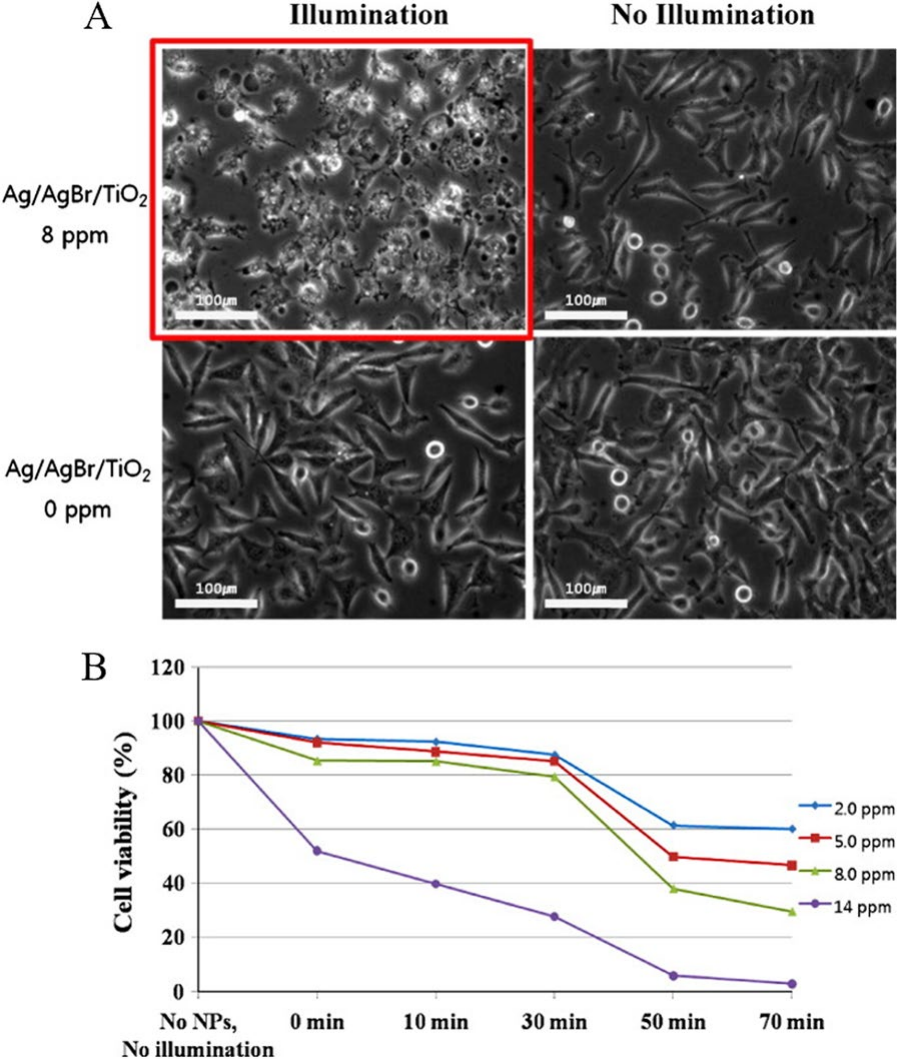
**A** Optical inverted phase-contrast microscopy images for the viability of HeLa cells treated with Ag/AgBr/TiO_2_ NPs incubated for longer than 24 h. Viability of HeLa cells as a function of halogen light illumination time. **B** The trypan blue exclusion test. The cells were treated with different Ag/AgBr/TiO_2_ NPs concentrations in the range of 2.0, 5.0, 8.0, and 14.0 ppm. Adapted from Ref. [112] with permission. Copyright (2011), Journal of Hazardous Materials

In 2011, Seo’s group reported that serum protein-adsorbed Ag/AgBr/TiO_2_ NPs have photocytotoxicity in vitro and in vivo under visible light [112]. In this case, both Ag/AgBr and TiO_2_ had photocatalytic activities. The authors prepared Ag/AgBr/TiO_2_ by the deposition–precipitation method and found that they appeared to be well internalized by human carcinoma cells. As shown in Fig. 3A, the authors tested two types of cancer cells including human cervical HeLa and skin A431 cancer cells after incubation with and without the Ag/AgBr/TiO_2_ and subsequent exposure to visible light from a halogen lamp. Fluorescence images were taken to evaluate the labeled mitochondria activity, and the results suggested that ROS triggered the photo-destruction of the cancer cells. After applying the halogen light illumination for 50–250 min and ∼8 ppm (μg/mL) of the photocatalytic Ag/AgBr/TiO_2_, the authors observed a 40–60% decrease in cell viability (Fig. 3B). Finally, the Ag/AgBr/TiO_2_ was found to eliminate xenograft tumors significantly by irradiating visible light in vivo for 10 min.

**Fig. 4:**
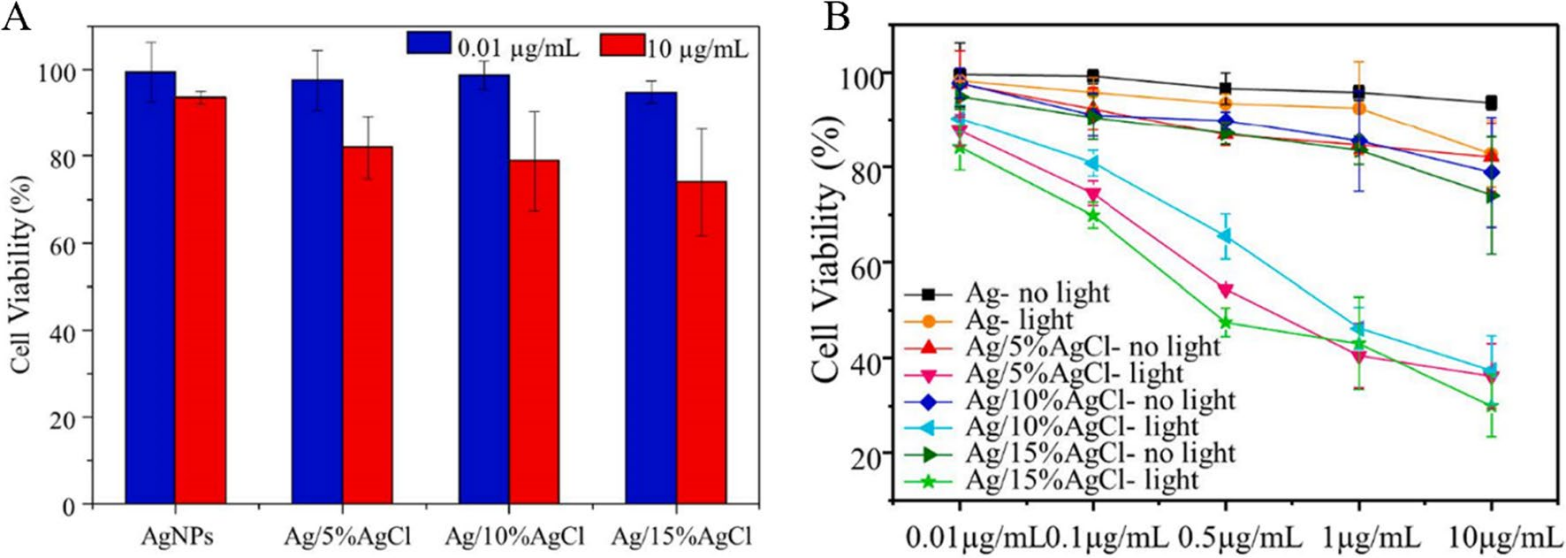
**A** The cell viability of Ag and different ratios of AgCl at Ag treated HeLa cells in a dark environment. **B** The comparison of HeLa cell viability treated by Ag and Ag/AgCl with or without simulated sunlight. Adapted from Ref. [68] with permission. Copyright (2022), Chemical Engineering Journal

The above two examples had either DNA or TiO_2_ on the surface of Ag/AgX. In 2021, Zhang et al. reported that naked Ag/AgCl NPs can be applied for photocatalytic cancer treatment [68]. In this work, the authors synthesized biocompatible and surfactant-free Ag/AgCl NPs to investigate the photocatalytic efficacy. AgCl can be oxidized by FeCl_3_ on the surface of AgNPs, which naturally formed a Schottky junction. Thus, Ag as a co-catalyst can easily trap the photo-generated charge carriers and then provide free electrons, which would decrease the influence of the recombination process and increase photocatalytic efficacy. The photocatalytic efficacy of the Ag/AgCl NPs eliminated over 75% of HeLa cells under 30 min of light irradiation (Fig. 4A). Besides that, light irradiation stimulated the SPR of the AgNPs, which can enhance visible light absorption by the material. Compared to the pure AgNPs, the AgCl-coated AgNPs had a broader range of working wavelengths and higher photocatalytic efficacy (Fig. 4B). Moreover, it has less dark cytotoxicity than the pure AgCl NPs.

### Cancer Cell Detection

An early cancer diagnosis is critical for the treatment and survival of cancer patients. Its purpose is to detect and treat early, and to reduce the physical, mental and economic burden of patients. Early cancer physical examination methods include a series of new cancer physical examination methods such as blood drop detection, genetic testing, nano-detection, and imaging [113, 114]. Laboratory tests can be used to analyze tumor markers, which substances are higher than normal levels found in the blood, urine, or tissues of some cancer patients. Imaging tests used to diagnose cancer may include computed tomography (CT) scans, bone scans, magnetic resonance imaging (MRI), positron emission tomography (PET) scans, ultrasound, and X-rays.

The methods for cancer cell detection can be conducted in two simple approaches. The first is that Ag/AgX nanomaterials can be composited with specific aptamers for cancer cells or folic acid (FA). Then, tumor-specific antigens or folate receptors will attract and bind the nanomaterials on those tumor cells. After that, colorless TMB is added in vitro and in vivo, which can be oxidized by Ag/AgX nanomaterials under light irradiation to blue-colored oxTMB, which can stain the tumor area. The second approach takes advantage of a high level of glutathione (GHS) in the tumor microenvironment, which has been generally regarded as a characteristic of cancerous tissues. AgBr nanomaterials can react with glutathione (GHS) to form AgNPs, which have a strong photoacoustic (PA) signal in the NIR-II region.

Silver halide has excellent imaging capability based on its physical property, which can be used in cancer detection. In 2014, Wang et al. reported a series of chitosan (CS) modified AgX NPs, which had dual-responsive enzyme mimetic activities [115]. In the presence of H_2_O_2_, the CS/AgX NPs were able to oxidize various colorimetric dyes, namely, peroxidase-like activity. Upon photoactivation, CS-AgX NPs could also oxidize the typical substrates in the absence of H_2_O_2_. Thus, the CS-modified silver halide was found to have dual-responsive enzyme mimetic activities. Based on these findings, the CS-AgI was regarded as a cost-effective, rapid, and highly sensitive colorimetric assay to detect cancer cells under light irradiation. The detection limit of the method for MDA-MB-231 was estimated to be as low as 100 cells, which was much lower than that reported by the method using peroxidase mimicking nanozymes based on other nanomaterials [116, 117].

**Fig. 5:**
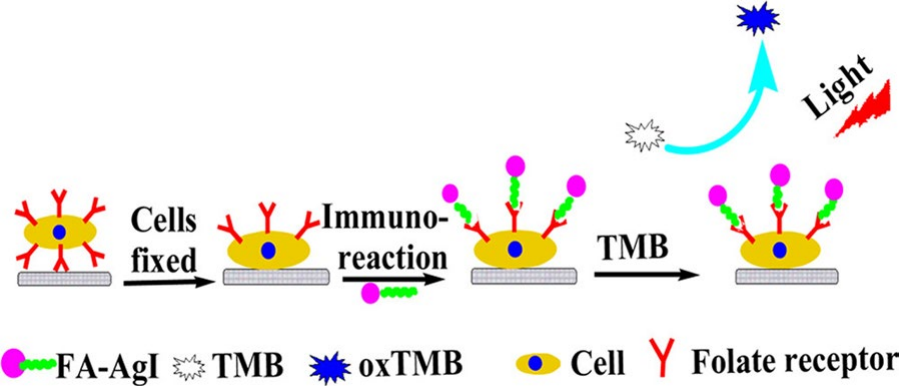
Proposed detection process using FA-CS-AgI. Adapted from Ref. [115] with permission. Copyright (2014) ACS Publications

Folic acid (FA), one of the best-characterized biological ligands, is widely employed to target tumors or cancer cells because folate receptors are usually overexpressed on the membrane of human cancer cells [118, 119]. FA-CS-AgI conjugates were synthesized by chemically coupling FA to CS via the formation of an amide bond between the amine groups of CS and the carboxyl groups of FA. As Fig. 5 shows, cells were first fixed on a 96-well plate. Then the FA-AgI conjugate was added to the folate receptor, which is a simple binding reaction and no antibodies were involved. Then, taking advantage of the photo-oxidase-like activity of AgI, TMB was added as a substrate. Upon irradiation with visible light (*λ* ≥ 420 nm), blue-colored TMB oxidation products were formed in the presence of tumor cells.

**Fig. 6:**
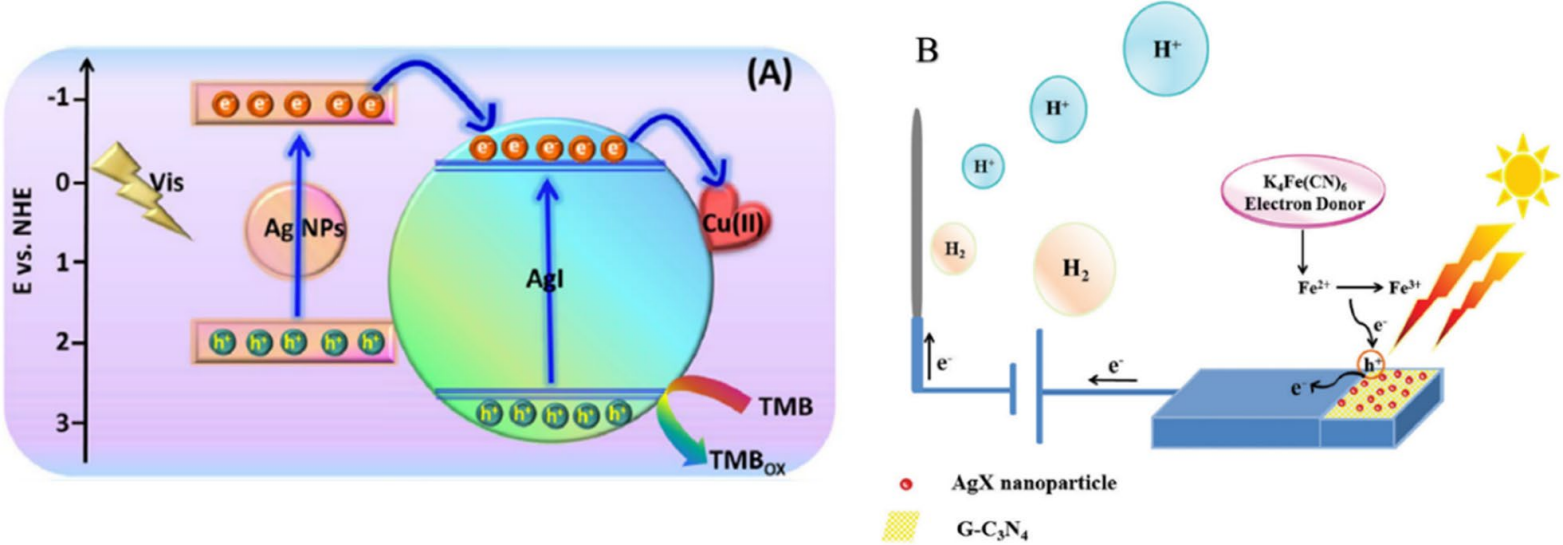
**A** The schematic illustration of oxidation of TMB by Cu^2+^/Ag–AgI **B** Photo-generated electron–hole transfer mechanism of C_3_N_4_–AgX in the presence of 0.1 M K_4_[Fe(CN)_6_]. Adapted from Ref. [120] with permission. Copyright (2017), Talanta. Copyright (2018), Journal of Materials Chemistry B

In 2017, Zhang’s group reported a Cu^2+^-doped Ag–AgI nanocomposite (Cu^2+^/Ag–AgI), which was designed for photosensitive colorimetric immunoassay for tumor marker detection [120]. Figure 6A clearly shows that it was designed as a highly photosensitive colorimetric immunoassay for carcinoembryonic antigen (CEA) detection based on the Cu^2+^/Ag–AgI. The Cu^2+^/Ag–AgI can oxidize TMB, which was synthesized by an impregnation method. After that, they achieved the immune-complex built on the surface of a magnetic bead by utilizing Cu^2+^/Ag–AgI as labels based on a sandwich-type immunoassay. The blue color of TMB_ox_ was obtained under visible irradiation and ultraviolet spectrum scanning, which showed increased absorbance values with increasing CEA concentrations. Above all, the developed colorimetric immunoassay exhibited good selectivity, repeatability, stability, and possible applications in the real serum sample analysis.

In 2018, Mazhabi et al. reported a C_3_N_4_–AgI/ITO photoelectrode for specific cervical cancer HeLa cell recognition [121]. They claimed that the C_3_N_4_–AgI/ITO photoelectrode as a label-free aptamer-based cytosensor can be applied in cancer diagnostics. As a new nanomaterial, the C_3_N_4_–AgI/ITO photoelectrode has both photoactive and bio-recognition features, which perfectly satisfied the requirements of photoelectrochemical (PEC) biosensors for cancer cell detection. In this study, the authors designed a novel label-free PEC aptamer-based cytosensor for the specific detection of cancer cells such as HeLa cells by using water-dispersible g-C_3_N_4_–AgI nanocomposites as visible light-sensitive materials and an anti-CEM/PTK7 aptamer as the bio-recognition element. As shown in Fig. 6B, when a suitable amount of AgI NPs was doped in the two-dimensional graphite-like carbon nitride nano-sheets (g-C_3_N_4_ NSs), the visible light photocurrent response could be significantly improved. The PEC response of the as-prepared biosensor based on the g-C_3_N_4_–AgI/ITO photoelectrode was linearly proportional to the relevant cancer cells such as HeLa cells in concentrations ranging from 10 to 10^6^ cells per mL with a limit of detection of 5 cells per mL. In addition, the g-C_3_N_4_–AgI/ITO photoelectrode and the fabricated cytosensor exhibited long-term stability, good reproducibility, excellent selectivity, and high sensitivity, demonstrating the successful conjugation of g-C_3_N_4_–AgI NSs with the aptamer and targeting cancer cells in the high-performance PEC cytosensor.

**Fig. 7:**
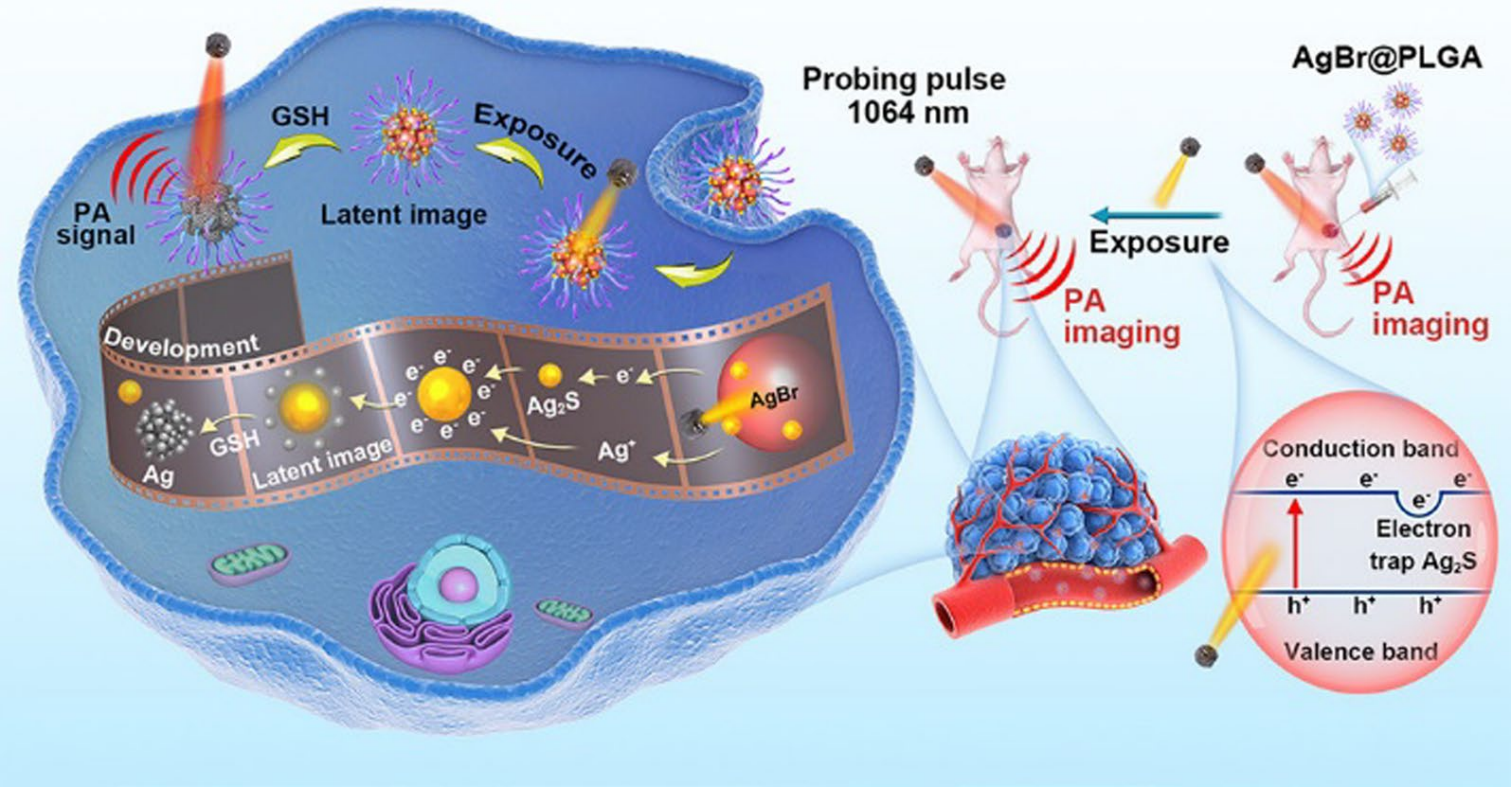
Synthesis process of the prepared AgBr@PLGA NCs and the schematic illustration that tumor area is graphically fixed via redox reaction where the NIR-transparent AgBr@PLGA NCs can be reduced by the tumor-abundant GSH into strongly absorbing silver NPs. Adapted from Ref. [122] with permission. Copyright (2021), ACS Publications

In 2021, Cui et al. reported that AgBr@PLGA nanocrystals can be applied in ultrahigh-sensitive and tumor-specific photoacoustography in the near-infrared NIR-II region [122]. The highly up-regulated glutathione (GSH) concentration in the tumor microenvironment is generally identified to be an effective endogenous characteristic of cancerous tissues. Therein, an ultrahigh-sensitive and tumor-specific photoacoustography technique in the NIR-II region based on optical writing and redox-responsive chromogenic graphic fixing was developed by introducing a self-synthesized photosensitive silver bromide modified with poly lactic-co-glycolic acid (AgBr@PLGA) nanocrystals. After optically triggered by external light, the NIR-transparent AgBr@PLGA nanocrystals can be reduced by the tumor-abundant GSH into strongly absorbing AgNPs, significantly boosting the “turn-on” photoacoustic (PA) signal in the NIR-II region. As shown in Fig. 7, the tumor area can be graphically fixed and developed in the photoacoustography. Experiments on both in vitro phantoms and in vivo mouse models demonstrated that the tumor area was specifically identified by the photoacoustography with the background signals effectively suppressed by dynamically modulating the exposure time.

**Fig. 8:**
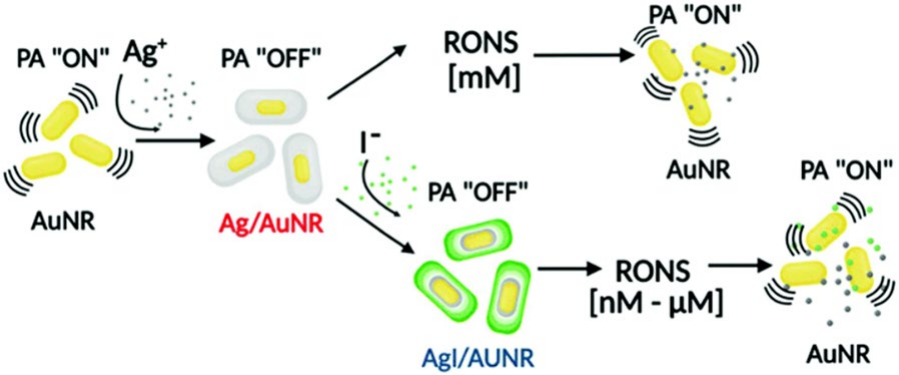
Schematic of shell optimization. Schematic representation of the current work. Iodide doping results in biologically relevant [nM–μM] sensitivity to RONS. Adapted from Ref. [123] with permission. Copyright (2020), Royal Society of Chemistry

Mantri et al. reported that iodide-doped precious metal NPs, such as Ag@AuNR, can be used to measure oxidative stress in vivo via photoacoustic imaging [123]. Since the accumulation of reactive oxygen and nitrogen species (RONS) can directly influence cell damage and cell death, the measurement of RONS is important for cancer detection. However, both reactive oxygen and nitrogen species are short-lived and exist only in trace amount in vivo (biologically RONS levels are in the nM–μM scale). Therefore, they used halide doping to tune the electrochemical properties of gold-core/silver-shell NPs to match the oxidation potential of RONS. The proposed mechanism is shown in Fig. 8. In this work, the authors provided the synthesis, characterization, and application of these AgI-coated gold nanorods (AgI/AuNR) and discussed that halide doping lowered the reduction potential of Ag, which resulted in a 1000-fold increase in H_2_O_2_ and 100,000-fold increase in ONOO^−^ sensitivity. The AgI/AuNR system also etched 45-fold faster than the undoped Ag/AuNR.

## AgX-Based Antibacterial Nanomaterials

Bacteria are an important cause of disease and infection. Some microorganisms such as golden staphylococci may cause serious human tissue damage. Thus, the preparation of antibacterial materials has caused widespread interest [6, 124, 125]. One of the well-known applications of AgNPs is antimicrobial. Aside from pure AgNPs, using its AgX hybrid materials has also been reported.

Recently, Ag@AgCl as a highly efficient photocatalyst was applied to the photocatalytic degradation of pollutants and organic synthesis products [63, 126–128]. Since the charge hole combination rate of the AgCl is low under visible light, and the active oxygen species, such as hydroxyl radicals, are relatively high in concentration and are applied to the preparation of antibacterial materials.

**Fig. 9:**
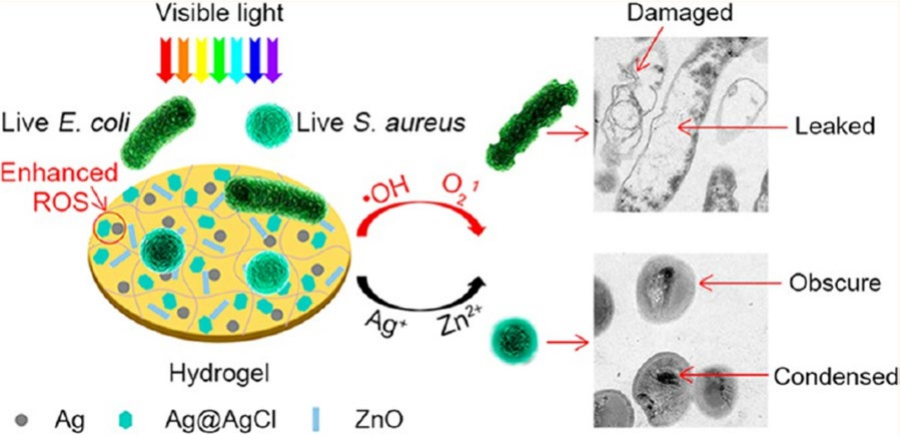
Mechanism of Ag/Ag@AgCl/ZnO antibacterial. Adapted from Ref. [8] with permission. Copyright (2017) ACS Nano

In 2017, Mao et al. reported that Ag/Ag@AgCl/ZnO hybrid nanostructures exhibited a high antibacterial efficiency against both *Escherichia coli* (*E. coli*) and Staphylococcus aureus under visible light irradiation (Fig. 9) [8]. In their study, the Ag/Ag@AgCl nanostructures enhanced the antibacterial activity of ZnO. This hydrogel system killed 95.95% of *E. coli* and 98.49% of S. aureus under visible light irradiation after 20 min. In addition, this system provided about 90% Zn^2+^ release in the acidic environment within 3 days, but 10% Zn^2+^ release occurred in the neutral environment within 21 days. The results showed that the release of Ag^+^ and Zn^2+^ stimulated the immune function to produce a large number of white blood cells and neutrophils, which resulted in the production of antibacterial effects.

Silver bromide (AgBr) is an n-type photosensitive semiconductor with a 2.6 eV bandgap [61]. Ag/AgBr NPs are applied to the disinfection of bacteria because of the plasma effect and high efficiency charge molecular efficiency under light irradiation [59, 129].

**Fig. 10:**
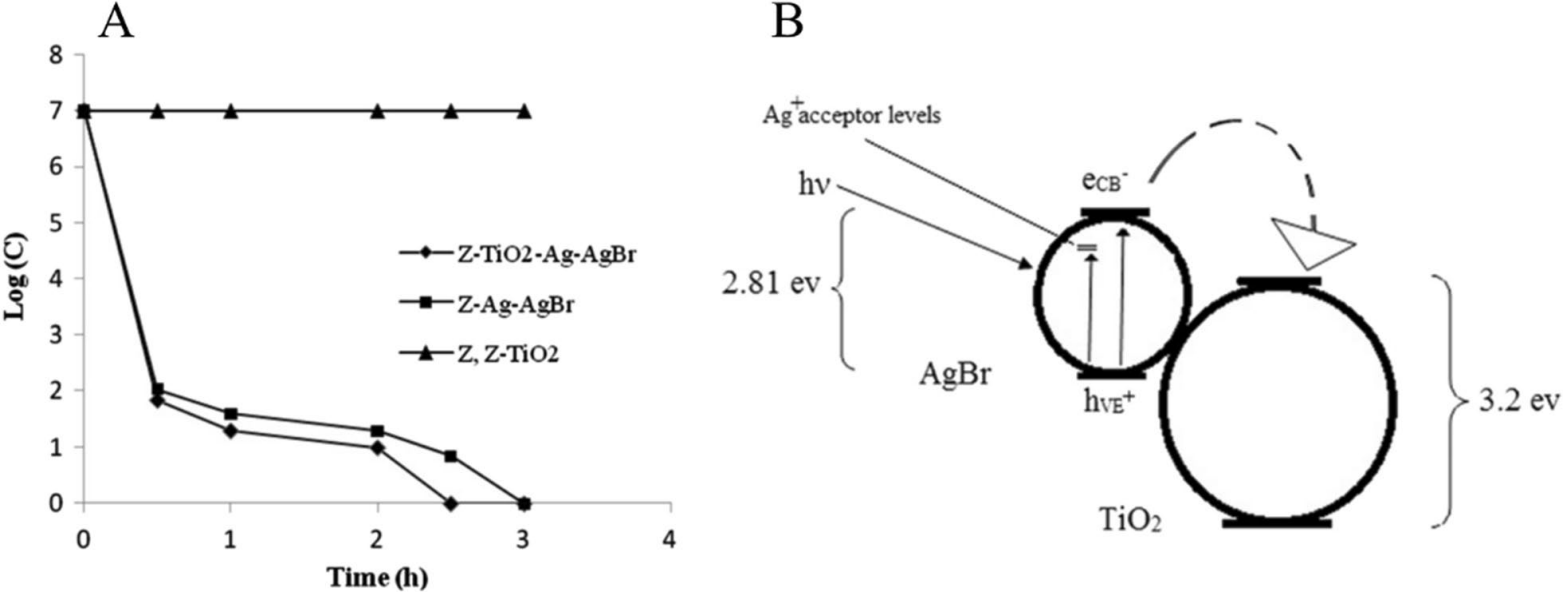
**A** *E. coli* inactivation curve over the composites at the visible light (C_0_ = 1 × 10^7^, 30 mL, 12 mg of catalyst). **B** Electrons transfer from AgBr conduction band to the lower position TiO_2_ conduction band and delay the electron–hole recombination. Adapted from Ref. [9] with permission. Copyright (2012) Materials Science in Semiconductor Processing

In 2012, Padervand et al. evaluated the photocatalytic activities of zeolite-based Ag/AgBr and Ag/AgBr/TiO_2_ photocatalysts for the inactivation of E. coli [9]. In their study, the Ag/AgBr/zeolite and Ag/AgBr/TiO_2_/zeolite had a high activity and decreased *E. coli* concentration most, but the zeolite and TiO_2_/zeolite had a lower antibacterial activity (Fig. 10A). After the visible light illumination, the electron–hole pairs of Ag/AgBr/TiO_2_/zeolite were generated. The electrons of AgBr conduction band can be transferred to TiO_2_ conduction band, which is below the AgBr conduction band. This property increased the photoreaction efficiency (Fig. 10B).

**Fig. 11:**
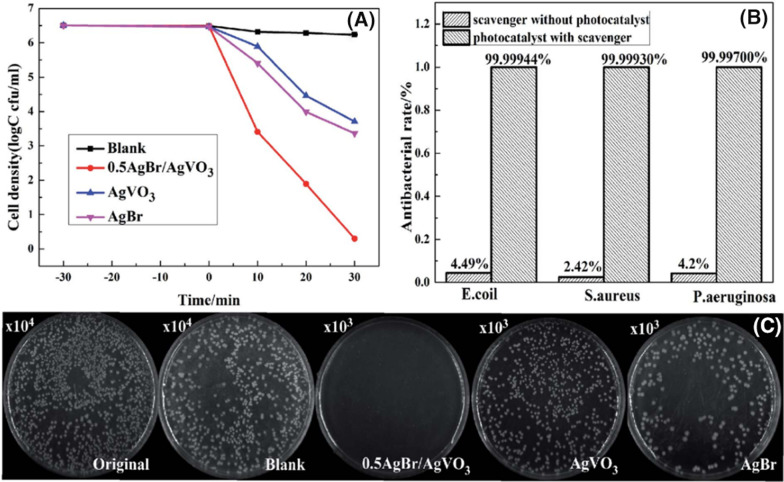
**A** *P. aeruginosa* survival curves in the antibacterial experiments, **B** photocatalytic antibacterial rates of *E. coli*, *S. aureus* and *P. aeruginosa* with 0.5AgBr/AgVO_3_ for 30 min, and the survival pictures of *P. aeruginosa* colonies in the presence of different photocatalysts. **C** Copyright (2019). Adapted from Ref. [130] with permission. The Royal Society of Chemistry

In 2019, Zhang et al. reported that AgBr/AgVO_3_ photocatalysts exhibited enhanced antibacterial efficiency compared to pure AgBr and pure AgVO_3_ under visible light irradiation [130]. More than 99.9970% of *E. coli*, *S. aureus*, and *P. aeruginosa* cells were killed by the photocatalysis of 0.5AgBr/AgVO_3_ after 30 min. Overall, the 0.5AgBr/AgVO_3_ heterojunction photocatalyst had excellent photocatalytic antibacterial performance (Fig. 11).

**Fig. 12:**
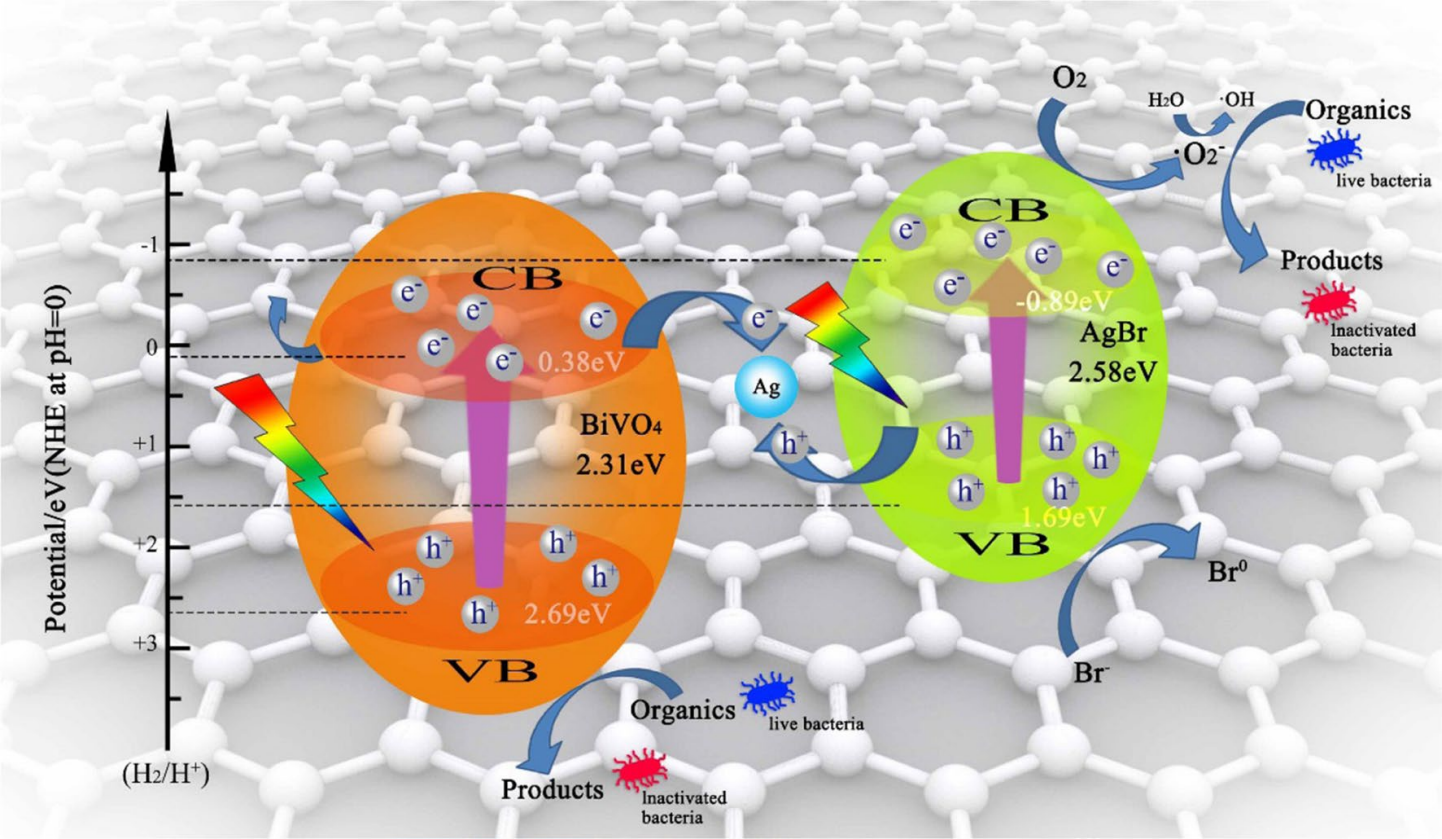
The proposed tentative mechanism for photocatalytic disinfection of Ag-AgBr/ BiVO_4_/GA composite under visible light irradiation. Adapted from Ref. [131] with permission. Copyright (2020) Colloids and Surfaces A

In 2020, Lin et al. reported a three-dimensional structured Ag-AgBr/BiVO_4_/graphene aerogel (Ag-AgBr/BiVO_4_/GA) via a hydrothermal and freeze-drying process [131]. In their study, the Ag-AgBr/BiVO_4_/GA exhibited a good disinfection performance toward *E. coli* (100% removal rate in 24 min). The proposed photocatalytic mechanism of inactivating *E. coli*/*S. aureus* by Ag-AgBr/BiVO_4_/GA is presented in Fig. 12. Ag-AgBr/BiVO_4_/GA was activated to excited states under visible light, and electrons in the valence band of AgBr and BiVO_4_ were excited to the conduction band (CB). Thus, both two semiconductors can generate electrons and holes. The existence of GA served as an electro-reservoir. Subsequently, the electrons formed in the CB of BiVO_4_ moved to the AgNPs, and the holes transferred from the VB of the AgBr, which caused the accumulation of electrons in the CB of AgBr and holes in the VB of BiVO_4_. Finally, the electrons in the CB reacted with O_2_ or H_2_O to generate active oxygen species, which can oxidize live bacteria into inactive bacteria.

**Fig. 13:**
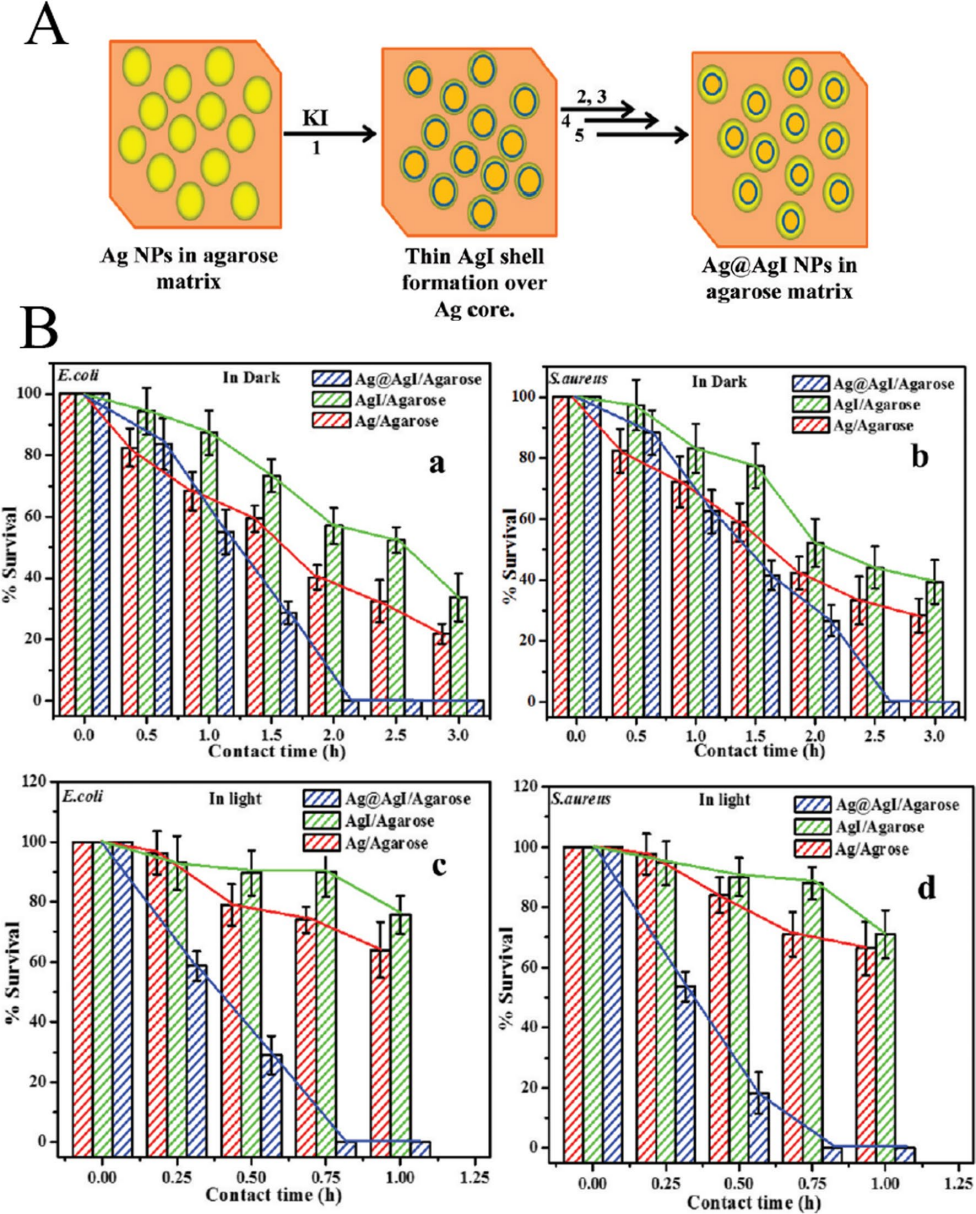
**A** Schematic illustration of the stepwise formation of core@shell structure of Ag@AgI in agarose matrix. **B** Viable cell count test by nutrient agar plating: percentage survival bacteria versus contact time for (a) *E. coli* and (b) *S. aureus* in the dark, and (c) *E. coli* and (d) *S. aureus* in the presence of light. Adapted from Ref. [7] with permission. Copyright (2012) Langmuir

Not only that but AgI can also be used as antibacterial material. In 2012, Ghosh et al. synthesized a core@shell structure consisting of AgNPs and AgI in an agarose matrix (Ag@AgI/agarose), which was an efficient antibacterial agent [7]. In situ synthesis of the catalyst was achieved by adding a low concentration of KI solution gradually. The authors speculated that the AgI thin layer was first formed by AgNPs, and then the AgI layer gradually became thick with the addition of the KI solution (Fig. 13A). Antibacterial studies including repetitive cycles were carried out on Gram-negative *E. coli* and Gram-positive Staphylococcus aureus bacteria in saline water, both in dark and under visible light. The Ag@AgI/agarose could be reused in the antibacterial experiment (Fig. 13B).

## AgX-Based Biosensors

A biosensor is a target-specific biomolecule coupled with a signal transduction unit that can be used to identify target analytes with high sensitivity and specificity. AgX has been used to prepare a high sensitivity biosensor, which can detect genes, urine, and blood in humans [132–135]. A recent development is the detection of glucose. Glucose oxidase can oxidize glucose to produce gluconolactone and the reduced enzyme. Subsequently, the reduced enzyme transfers electrons to molecular oxygen in the body's biological fluid to produce hydrogen peroxide (H_2_O_2_) [136, 137]. Ag/AgX as electrode materials were applied in several biosensor systems because it has good reversibility and its electrode potential is relatively stable [138].

The stability of biosensors is significant to real-world applications. Table 1 summarizes the durability of those Ag/AgX nanomaterials. Most of them used degradation efficiency and control of the light on/off states to test the stability of biosensors.

**Table 1:** The stability of Ag/AgX biosensors

Materials	Methods	Cycles/time	Performance decrease	References
GH/PANI/Ag@AgCl	Degradation efficiency	6 cycles	3%	[139]
AgI/Ag/BiOI	Switching the light source	15 cycles	0%	[140]
CS–AgI/TiO_2_	Degradation efficiency	4 cycles	0%	[48]
AgI/CBO/FTO	On/off illumination	5 min	0%	[141]
ITO/g–C_3_N_4_–AgI	On/off illumination	500 s	2%	[121]
AuNPs–AgCl@PANI	Reduction and oxidation of H_2_O_2_	7 months	Excellent catalytic effect	[142]

In 2021, Chen’s group reported a new nanomaterial, Ag@AgCl photocatalyst loaded on 3D graphene/PANI hydrogels that can be used for in situ SERS monitoring [139]. With silver halides AgX (X = Cl, Br, I) and the surface plasmon resonance (SPR) of metallic Ag, heterojunctions of Ag@AgX can exhibit high stability and highly efficient utilization of visible light. The authors synthesized GH/PANI/Ag@AgCl nanocomposites by reacting Ag^+^ ions with the Cl^−^ in the HCl-doped PANI in 3D GH/PANI and the subsequent photo-assisted reduction. These new nanomaterials have enhanced adsorption-photocatalytic degradation. Due to combining AgNPs with graphene and PANI, it can be applied for real-time SERS detection. Hence, graphene/PANI composites exhibited enhanced electrical conductivity and electrochemical stability compared with bare PANI, which has been widely applied in biosensors because of its optical, electronic, and electrochemical features.

**Fig. 14:**
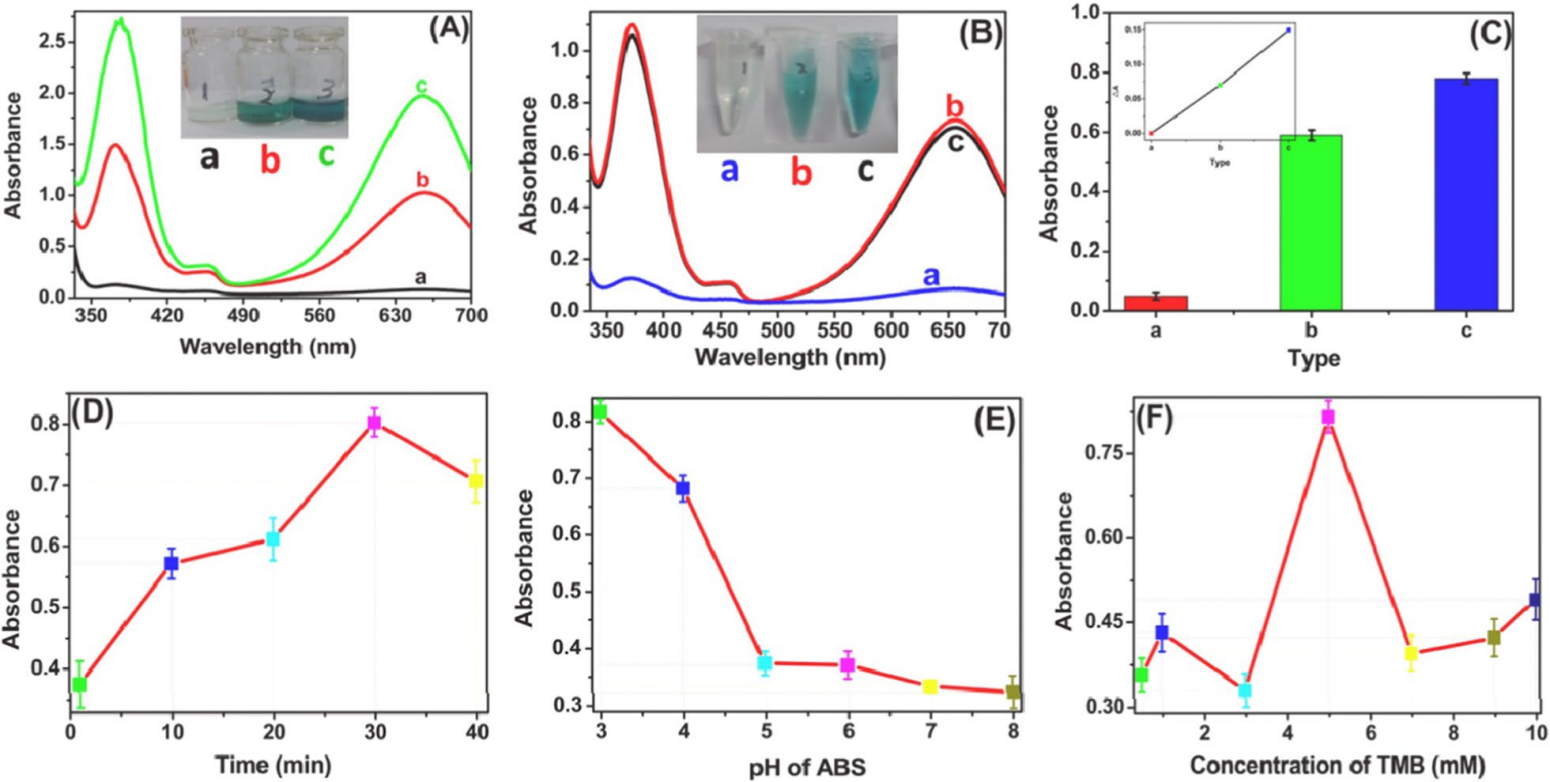
**A** The UV–Vis spectrums of (a) TiO_2_ + TMB, (b) AgI + TMB, and (c) CS-AgI/TiO_2_ + TMB under visible light illumination [the inset is the photographs of a, b, c]; **B** The UV–Vis spectrums of (a) Fe_3_O_4_ + TMB, (b) CS-AgI/TiO_2_ + TMB, and (c) Fe_3_O_4_ + CS–AgI/TiO_2_ + TMB; **C** the UV–Vis absorbance of different immunosensors for 0.5 nM CAP (a) anti-CAP-CS-TiO_2_/CAP-BSA/MB, (b) anti-CAP-CS-AgI/CAP-BSA/MB, and (c) anti-CAP-CS-AgI/TiO_2_/CAP-BSA/MB [the inset is ΔA of a, b, c for 0.5 nM CAP]; The effect of **D** incubation time of antigen–antibody, **E** pH of ABS, and **F** concentration of TMB. Adapted from Ref. [48] with permission. Copyright (2017) Biosensors and Bioelectronics

2016, Chang et al. used AgI/TiO_2_ hybrid material as a label to produce a color signal with a visible optic excitation for quantitative analysis of chloramphenicol (CAP) (Fig. 14). [48] The enzyme-like catalytic nanomaterials are also known as nanozymes [143–146]. Using a competitive immunoassay with CS-AgI/TiO_2_ labeled CAP antibodies as tags, detection of CAP was achieved on the surface of CAP-BSA modified magnetic bead. In this reaction, the participation of hydrogen peroxide was not required [48]. The combination of AgI and TiO_2_ effectively reduced the composite of photocarriers and electrons, which greatly enhanced the ability of solution color (Fig. 14A,B). This allowed the immunosensor sensor to have better sensitivity and a low detection limit of 0.03 nM.

**Fig. 15:**
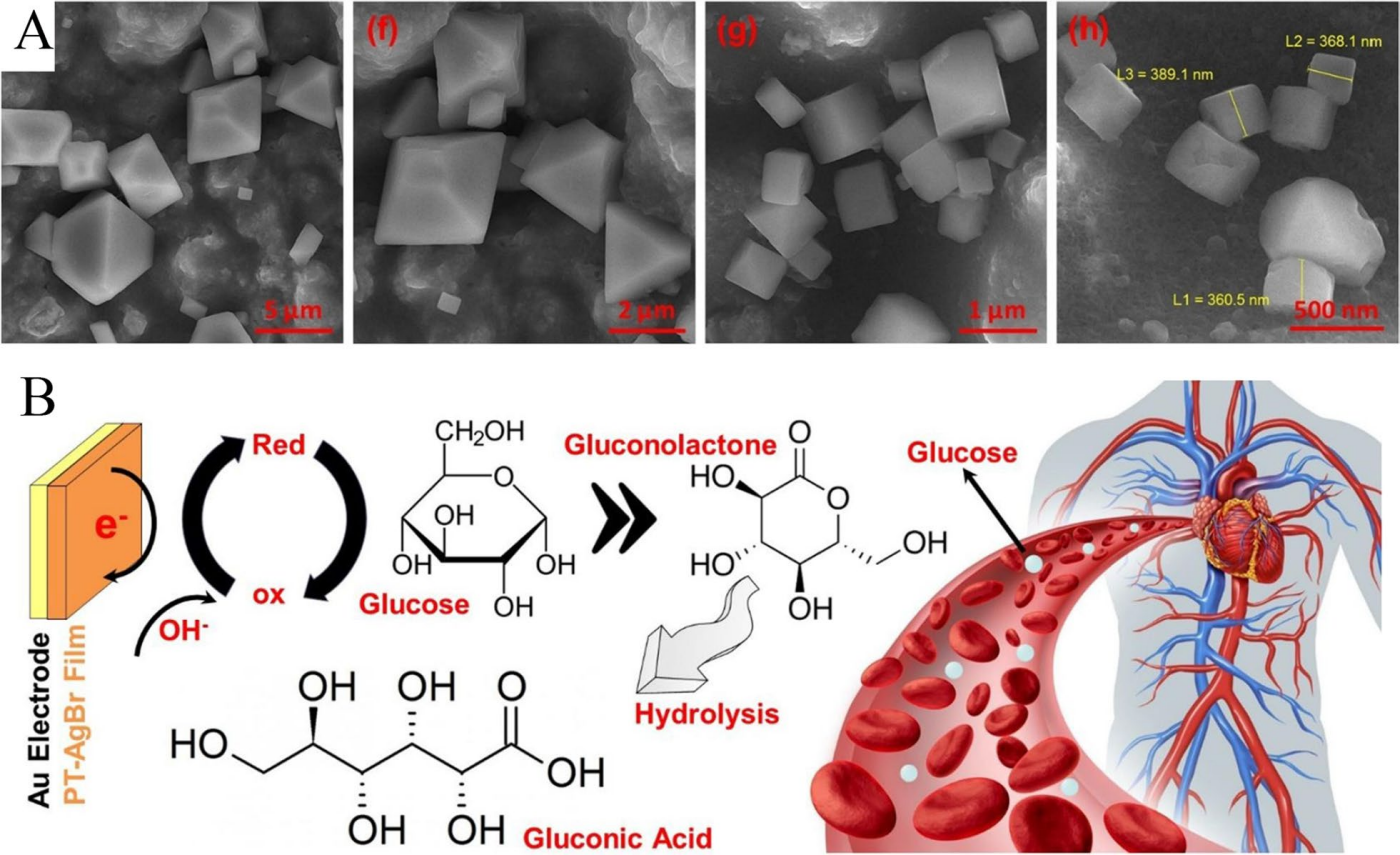
**A** FESEM images of (e–h) PT-AgBr **B** Non-enzymatic biosensing mechanism of glucose via the developed platform within the human blood plasma. Adapted from Ref. [138] with permission. Copyright (2020) European Polymer Journal

In 2020, Hashemi et al. reported that AgBr NPs with the polymeric structure of PT could sense glucose through a real-time and precise approach [138]. In their system, PT-AgBr was modified cubic, anorthic or hybrid cubic–anorthic morphologies (Fig. 15A). They confirmed the successful formation of AgBr, PT and PT-AgBr by diverse characterizations, for example, X-ray diffractogram, zeta potential, and FESEM. PT has a conductive polymeric structure. So, its integration with AgBr can improve its electrocatalytic performance, Further, glucose detection can be accurately performed (Fig. 15B), and the PT-AgBr showed zeta potential and electrophoretic mobility of 16.7 mV/0.000129 cm^2^/Vs, respectively.

**Fig. 16:**
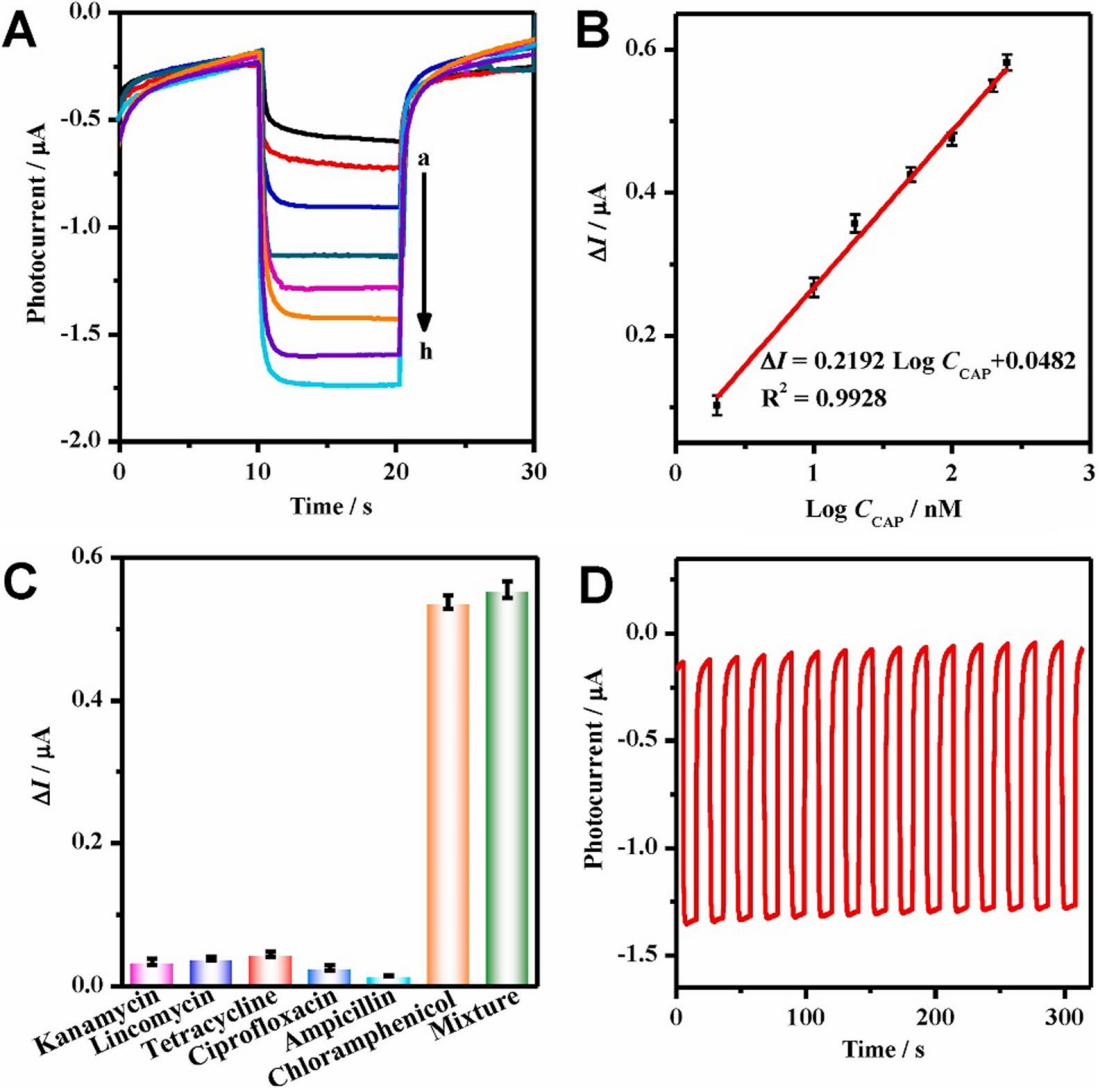
Photocurrents **A** of the developed PEC aptasensor with different CAP concentrations (curves a–h: 0–250 nM). Calibration curve (**B**). Selectivity (**C**) toward 200 nM CAP with equal amount of the interfering substances (i.e., kanamycin, lincomycin, tetracycline, ciprofloxacin, and ampicillin), and their mixture. Stability (**D**) of the as-fabricated PEC aptasensor. Adapted from Ref. [140] with permission. Copyright (2021) Biosensors and Bioelectronics

In 2021, Zhu et al. reported that 3D Z-scheme AgI/Ag/BiOI heterojunction for highly sensitive determination of CAP, thereby fabricating a “signal-on” PEC aptasensor [140]. In their study, the AgI/Ag/BiOI photocatalyst was prepared by an ion-exchange and photo-irradiation method, which improved the photocurrents by facilitating the spatial separation of the photo-generated charge carriers. The response of the PEC aptasensor was linearly correlated between the photocurrents and the logarithm of the CAP concentrations, which had the advantages of being sensitive, simple and stable (Fig. 16).

**Fig. 17:**
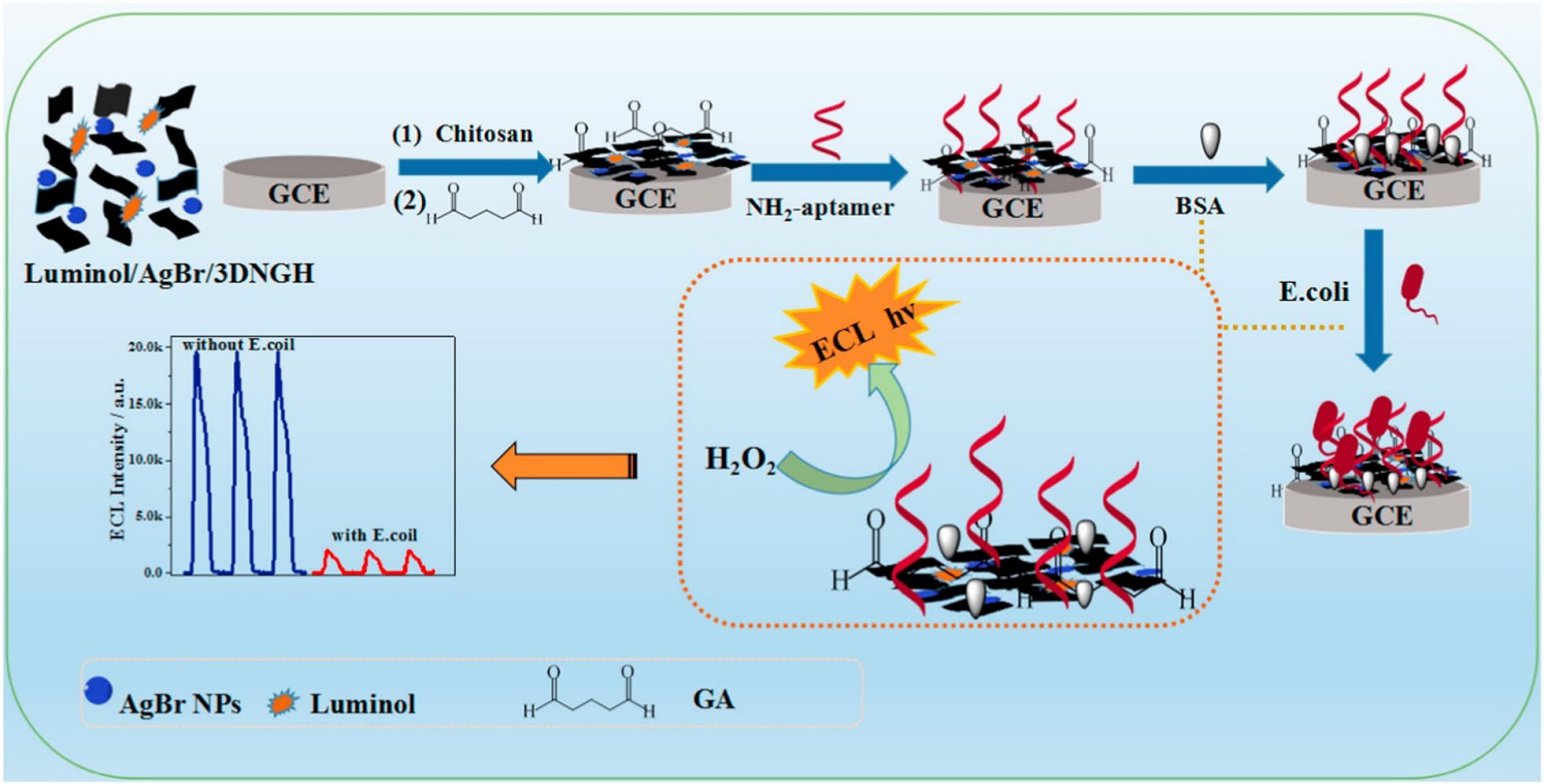
The schematic presentation for the ECL *Escherichia coli* biosensor fabricated with luminol/AgBr/3DNGH. Adapted from Ref. [147] with permission. Copyright (2017) Biosensors and Bioelectronics

In 2019, Hao et al. reported AgBr NPs-anchored 3D nitrogen-doped graphene hydrogel (3DNGH) nanocomposites with a large surface by hydrothermal method (Fig. 17) [147]. With a porous structure, 3DNGH had a large surface area that can favor the immobilization of AgBr and luminol, where in turn, luminol and hydrogen peroxide can be in close contact. They fabricated all-solid-state luminol electrochemiluminescence *E. coli* aptasensors, which showed great performance. So, the potential for the luminol/AgBr/3DNGH nanocomposite for E. coli detection in biomedical and environmental analysis was developed.

## Summary and Perspectives

In this article, we reviewed the biomedical and bioanalytical applications of AgX. Silver halide has been widely used in industry as electrodes (Ag/AgCl), imaging materials (AgBr), and artificial rainfall (AgI).

However, AgX NPs as photocatalytic materials have not been intensively studied because they decompose to produce AgNPs under visible light. Through the combination of silver NPs and silver halide, the decomposition rate of silver halide can be significantly reduced. The principle is that silver halide, as a semiconductor inorganic photocatalyst, will generate photo-generated carriers and generate electrons and holes under visible light irradiation. However, the rate of this process is low because electrons and holes will combine, reducing the occurrence of oxidation reactions. Therefore, the silver elementary substance can be doped into silver halide, because elemental silver, as a precious metal, can produce a surface plasmon effect and promote electron transport. One of the drawbacks is the potential photofatigue effect during long-term irradiation, which may result in a gradual loss of efficiency. The solution is that AgNPs and AgX form a Schottky junction, which reduces the contact between electrons and holes, thereby promoting photocatalytic efficiency and extending the working life. Based on the above discussion, it is possible to apply Ag/AgX photocatalysts in the fields of cancer treatment, cancer diagnosis, antibacterial, wound healing, and biosensor.

The applications of Ag/AgX in medicine are only at the proof-of-concept stage because the working wavelength for Ag/AgX nanomaterials was almost exclusively in the UV/visible region, and the short-wavelength light can suffer from phototoxicity and poor tissue penetration. To solve this problem, a near-infrared (NIR) light featuring lower toxicity and deeper tissue penetration has more advantages in biological applications. By doping heterojunctions and other methods, the range of photocatalytic wavelengths can be increased, and the energy bandgap can be reduced so that the silver halide nanomaterial can absorb more energy in a broad wavelength range. At the same time, AgX materials also have certain limitations. For example, simple AgX is susceptible to decomposition under strong light, and AgNPs produced by the decomposition have cytotoxicity to human tissues and organs. Therefore, further research on the application of silver halide surface plasma materials in the field of photocatalysis is indispensable.

## Data Availability

The datasets used or analyzed during the study are available from the related journals and the corresponding author upon reasonable request.
